# The fruit glossiness locus, *dull fruit* (*D*), encodes a C_2_H_2_-type zinc finger transcription factor, CsDULL, in cucumber (*Cucumis sativus* L.)

**DOI:** 10.1093/hr/uhac146

**Published:** 2022-07-02

**Authors:** Xuling Zhai, Haoying Wu, Yaru Wang, Zhongren Zhang, Li Shan, Xi Zhao, Ruijia Wang, Chang Liu, Yiqun Weng, Ying Wang, Xingwang Liu, Huazhong Ren

**Affiliations:** Engineering Research Center of the Ministry of Education for Horticultural Crops Breeding and Propagation, College of Horticulture, China Agricultural University, Beijing 100193, China; Beijing Key Laboratory of Growth and Developmental Regulation for Protected Vegetable Crops, College of Horticulture, China Agricultural University, Beijing 100193, China; Engineering Research Center of the Ministry of Education for Horticultural Crops Breeding and Propagation, College of Horticulture, China Agricultural University, Beijing 100193, China; Beijing Key Laboratory of Growth and Developmental Regulation for Protected Vegetable Crops, College of Horticulture, China Agricultural University, Beijing 100193, China; Engineering Research Center of the Ministry of Education for Horticultural Crops Breeding and Propagation, College of Horticulture, China Agricultural University, Beijing 100193, China; Beijing Key Laboratory of Growth and Developmental Regulation for Protected Vegetable Crops, College of Horticulture, China Agricultural University, Beijing 100193, China; Engineering Research Center of the Ministry of Education for Horticultural Crops Breeding and Propagation, College of Horticulture, China Agricultural University, Beijing 100193, China; Beijing Key Laboratory of Growth and Developmental Regulation for Protected Vegetable Crops, College of Horticulture, China Agricultural University, Beijing 100193, China; Engineering Research Center of the Ministry of Education for Horticultural Crops Breeding and Propagation, College of Horticulture, China Agricultural University, Beijing 100193, China; Beijing Key Laboratory of Growth and Developmental Regulation for Protected Vegetable Crops, College of Horticulture, China Agricultural University, Beijing 100193, China; Engineering Research Center of the Ministry of Education for Horticultural Crops Breeding and Propagation, College of Horticulture, China Agricultural University, Beijing 100193, China; Beijing Key Laboratory of Growth and Developmental Regulation for Protected Vegetable Crops, College of Horticulture, China Agricultural University, Beijing 100193, China; Engineering Research Center of the Ministry of Education for Horticultural Crops Breeding and Propagation, College of Horticulture, China Agricultural University, Beijing 100193, China; Beijing Key Laboratory of Growth and Developmental Regulation for Protected Vegetable Crops, College of Horticulture, China Agricultural University, Beijing 100193, China; Engineering Research Center of the Ministry of Education for Horticultural Crops Breeding and Propagation, College of Horticulture, China Agricultural University, Beijing 100193, China; Beijing Key Laboratory of Growth and Developmental Regulation for Protected Vegetable Crops, College of Horticulture, China Agricultural University, Beijing 100193, China; USDA-ARS, Vegetable Crops Research Unit, Horticulture Department, University of Wisconsin, 1575 Linden Dr., Madison, WI 53706, USA; Heze Agricultural and Rural Bureau, 1021 Shuanghe Road, Mudan District, Heze, Shandong, 274000, China; Engineering Research Center of the Ministry of Education for Horticultural Crops Breeding and Propagation, College of Horticulture, China Agricultural University, Beijing 100193, China; Beijing Key Laboratory of Growth and Developmental Regulation for Protected Vegetable Crops, College of Horticulture, China Agricultural University, Beijing 100193, China; Engineering Research Center of the Ministry of Education for Horticultural Crops Breeding and Propagation, College of Horticulture, China Agricultural University, Beijing 100193, China; Beijing Key Laboratory of Growth and Developmental Regulation for Protected Vegetable Crops, College of Horticulture, China Agricultural University, Beijing 100193, China

## Abstract

Fruit glossiness is an important external fruit quality trait for fresh-consumed cucumber fruit, affecting its marketability. Dull fruit appearance is mainly controlled by a single gene, *D* (for *dull fruit*) that is dominant to glossy fruit (*dd*), but the molecular mechanism controlling fruit glossiness is unknown. In the present study, we conducted map-based cloning of the *D* locus in cucumber and identified a candidate gene (*Csa5G577350*) that encodes a C_2_H_2_-type zinc finger transcription factor, CsDULL. A 4895-bp deletion including the complete loss of *CsDULL* resulted in glossy fruit. *CsDULL* is highly expressed in the peel of cucumber fruit, and its expression level is positively correlated with the accumulation of cutin and wax in the peel. Through transcriptome analysis, yeast one-hybrid and dual-luciferase assays, we identified two genes potentially targeted by CsDULL for regulation of cutin and wax biosynthesis/transportation that included *CsGPAT4* and *CsLTPG1*. The possibility that CsDULL controls both fruit glossiness and wart development in cucumber is discussed. The present work advances our understanding of regulatory mechanisms of fruit epidermal traits, and provides a useful tool for molecular breeding to improve external fruit quality in cucumber.

## Introduction

Cucumber, *Cucumis sativus* L. (2*n* = 2*x* = 14) is an economically important vegetable crop cultivated globally with China as the leading producer, accounting for >70% of world production (FAOSTAT 2020, https://www.fao.org/faostat/). Cucumber fruits are consumed immature, either raw or processed (pickles). Cucumbers adapted to the two uses may be different in many morphological traits, such as fruit size, skin texture and color, fruit firmness, crispness, and taste quality [1, 2]. Among them, the fruit glossiness of fresh market cucumbers is an important trait affecting consumer acceptance and marketability. In many markets, glossy fruit is more popular than dull fruit for its bright and shiny appearance [3], which is also an import target in cucumber breeding.

Fruit glossiness has long been recognized as a simply inherited trait, with dull fruit dominant to glossy [4, 5]. Kooistra [6] assigned the gene symbol *D* for *dull fruit*. Two genetic mapping studies with molecular markers placed the *D* locus on cucumber chromosome 5 [7, 8]. More recently, Yang *et al*. [3] delimited the *D* locus in a 245-kb region defined by simple sequence repeat (SSR) markers SSR37 and SSR112 with 31 annotated genes. Early phenotypic linkage analysis in segregation populations found that *D* had strong linkage with other fruit epidermal traits, including uniform immature fruit color (*u*), small spine (*ss*), tuberculated (warty) fruit (*Tu*), palisade epidermis (*Pe*), and tender fruit (*te*) [9–11]. Two fruit skin texture-related traits, fruit skin netting (*H* for *heavy netting*), and fruit ribbing (*Fr*), were also found to be linked to *D* [8, 12]. Recent molecular mapping studies on these fruit epidermal features have shown that these genes are indeed clustered in a small region on cucumber chromosome 5 [13]. Interestingly, specific allele combinations of these tightly linked genes for fruit external appearances are characteristic of different cucumber market classes, which likely reflects long-term selection and breeding practices adapting to local environments, production systems, and processing requirements, as well as consumer needs [2, 14].

The genetic control of fruit glossiness is not complicated, but the understanding of it has become complex due to the existence of the *G* gene reported by Dong *et al*. [15], who found that the glossy fruit trait in cucumber was controlled by a single nuclear gene, *G*, with glossy being dominant to dull. More interestingly, the *G* locus for glossy fruit was also mapped in a 454-kb region on chromosome 5, close to the region where the *D* locus has been mapped [3]. This raises the question of whether the *G* and *D* genes actually belong to the same locus. In addition, previous studies have shown co-localization of the *D* and *Tu* loci [3, 16]. It is also of interest to understand the relationship of the two genes. However, neither *D* nor the *G* gene has been cloned.

The objective of this study was to understand the regulatory mechanisms for fruit glossiness controlled by the *D* locus. Here, we developed segregating populations from the cross between two near-isogenic cucumber inbred lines (NILs), 2073-1 (dull fruit) and 2073-2 (glossy fruit). Through bulked segregant analysis (BSA) and fine mapping, we identified a candidate gene for the *D* locus that encodes a C_2_H_2_ zinc finger transcription factor (CsDULL). Through RNA-seq and protein–DNA interaction studies, we identified two potential target genes for CsDULL that are involved in regulation of cutin and wax biosynthesis/transportation.

## Results

### Phenotypic characterization of the *dull* mutant

**Figure 1:**
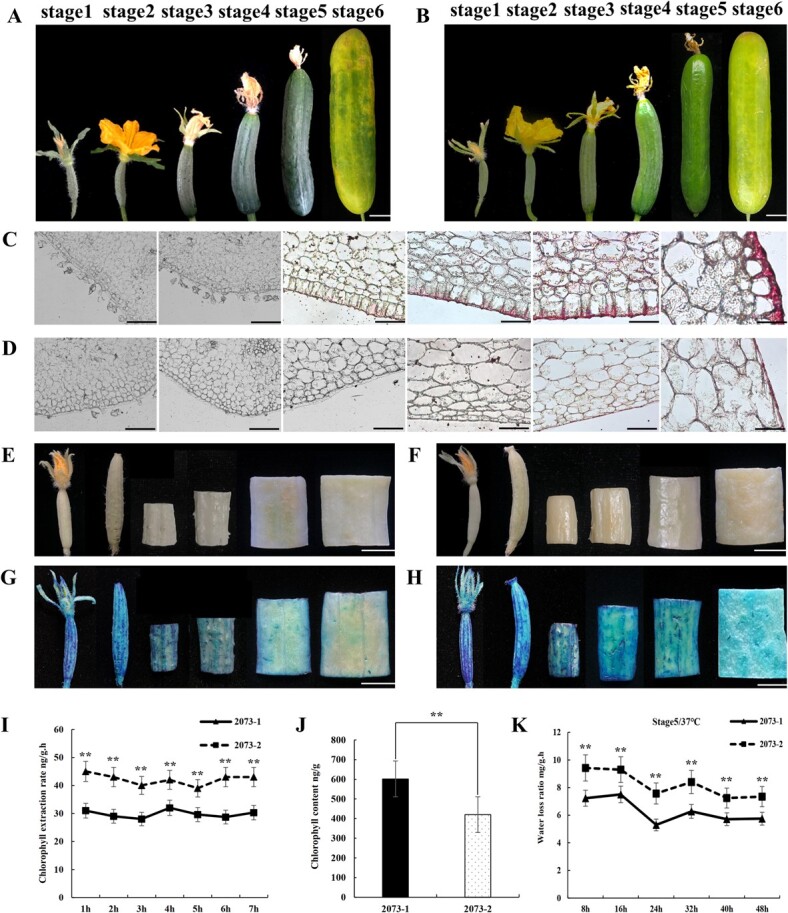
Phenotypic characterization of dull (2073-1) and glossy (2073-2) NILs at different development stages. (A, B) Fruit appearance of 2073-1 (A) and 2073-2 (B) at stages 1–6, corresponding to 5 days before pollination and 0, 5, 10, 15, and 30 days after pollination, respectively. (C, D) Sudan IV-stained ovaries/peels of 2073-1 (C) and 2073-2 (D). (E, F) Ovaries/fruit peels of 2073-1 (E) and 2073-2 (F) after decoloration. (G, H) Toluidine blue stained ovaries/peels of 2073-1 (G) and 2073-2 (H). In A–H, the images in each case are samples from stages 1 to 6 (left to right), respectively. (I) Chlorophyll leaching assays of peels at stage 5 from 2073-1 and 2073-2. Samples were placed in 85% ethanol and chlorophyll release was measured at the indicated times. Each data point is the mean ± standard deviation (*n* = 6). The double asterisks indicates a significant difference between 2073-2 and 2073-1 at *P* < .01 (*t*-test). (J) Total chlorophyll content of samples assayed in I and K. Water loss determinations of peels at stage 5 from 2073-1 and 2073-2. Samples were placed at 37°C and water loss ratio was measured at the indicated times. Each data point is the mean ± standard deviation (*n* = 6). The asterisk indicates a significant difference at *P* < .05 (*t*-test). Scale bar = 1 cm (A, B, E–H) or 250 μm (C, D).

The two parental lines, 2073-1 and 2073-2, are a pair of NILs at the *D* locus that bear dull and glossy fruits, respectively [17]. For convenience, based on the days before or after pollination we divided fruit development in the lines into six stages: stage 1 to stage 6. The fruits of 2073-1 showed the dull phenotype during the whole development process (Fig. 1A), whereas the fruits of 2073-2 showed a glossy phenotype from stage 2; this phenotype was most obvious at stage 5, close to commercial harvest (Fig. 1B). We also measured the glossiness (Gs) of both lines with a HYD-09 glossmeter at stage 5. The resulting data indicated that the Gs value of 2073-2 fruits was significantly higher than that of 2073-1, which was consistent with the visual rating (Supplementary Data Fig. S1).

Previous studies suggested that the glossiness of plant organs could be influenced by the chemical compositions and contents of epidermis cuticle [18–23]. We speculated that there may be defects in the cuticle of 2073-2 fruits. Therefore, we stained the fruits of 2073-1 and 2073-2 at different developmental stages using Sudan IV. We found that the cuticle of 2073-1 fruits already started lipid accumulation at stage 3, and accumulation accelerated with further fruit development (Fig. 1C). On the contrary, the cuticle of 2073-2 fruits had accumulated a very small amount of lipids at stage 4, and even stage 6 (Fig. 1D). This observation suggested that the cuticle of 2073-2 fruits is significantly defective, which may contribute to the glossy fruit phenotype.

The chemical composition of the cuticle may affect epidermal permeability. We measured the permeability of the fruit epidermis by toluidine blue (TB) staining, chlorophyll leaching assay, and water loss rate. To reduce the influence of chlorophyll on TB staining, we first decolored the samples with a mixture of acetone, absolute ethanol, and water (4.5:4.5:1) solution (Fig. 1E and F). No obvious visual difference was observed in TB-stained peels in stage 1 and stage 2 fruits of the two NILs (Fig. 1G and H). However, starting from stage 3, the peel of 2073-2 showed more small TB-stained spots than 2073-1; this was more obvious at stages 5 and 6, when the peel of 2073-1 was also slightly stained (Fig. 1G and H). The chlorophyll leaching assay revealed a significantly higher chlorophyll penetration rate of 2073-2 peel than that of 2073-1, even though 2073-2 had a lower chlorophyll content than 2073-1 (Fig. 1I and J). In addition, the water loss rate of 2073-2 fruits was also significantly higher than that of 2073-1 fruits (Fig. 1K).

### Inheritance of fruit glossiness

We investigated the inheritance mode of fruit glossiness using segregating populations (F_2_, BC_1_P_1_, and BC_1_P_2_) from crosses between 2073-1 (P_1_) and 2073-2 (P_2_) NILs. Phenotypic data for both parental lines and their F_1_, F_2_, and backcross progenies are presented in Table 1. All 2073-1 × 2073-2 F_1_ had dull fruit, like 2073-1 (Table 1). Segregation of dull to glossy in 2073-1 × 2073-2 F_2_ plants fitted a 3:1 ratio (*P* = .7418 in the *χ*^2^ test). All plants in the BC_1_P_1_ backcross population showed a dull phenotype while the ratio of dull to glossy plants in the BC_1_P_2_ population was 1.15:1 (122:106), which was consistent with 1:1 from the *χ*^2^ test (*P* = .2803) (Table 1). These data confirm earlier studies indicating that fruit glossiness is controlled by a single dominant gene, which is *D* according to the cucumber gene naming rule [14].

**Table 1:** Genetic analysis of the glossy phenotype.

**Lines/populations**	**Total**	**Dull**	**Glossy**	**Dull:glossy**	***P* (*χ*** ^**2**^ **test)**
2073-1 (P_1_)	26	26			
2073-2 (P_2_)	23		23		
P_1_ × P_2_ F_1_	146	146			
P_2_ × P_1_ F_1_	115	115			
(P_1_ × P_2_) F_2_	1486	1120	366	3.06:1	0.7418
BC_1_P_1_, (P_1_ × P_2_) × P_1_	216	216	0		
BC_1_P_2_, (P_1_ × P_2_) × P_2_	228	122	106	1.15:1	0.2803

### Genetic mapping and candidate gene identification for the *D* locus

To further refine the chromosomal location of the *D* locus, we performed BSA using the 2073-1 × 2073-2 F_2_ population. Equal amounts of genomic DNAs from 50 dull and 50 glossy plants were pooled to construct the dull and glossy bulks, respectively. The dull, glossy bulks and the 2073-1 and 2073-2 parental lines were subjected to Illumina high-throughput sequencing, from which 83.26, 76.13, 26.11, and 28.9 million paired-end reads were produced, representing 51×, 48×, 15×, and 17× genome coverage, respectively (Supplementary Data Table S1). Among them, 92.7, 91.4, 90.9, and 91.3% reads could be mapped to the reference genome, indicating good quality of the sequencing data (Supplementary Data Table S1).

**Figure 2:**
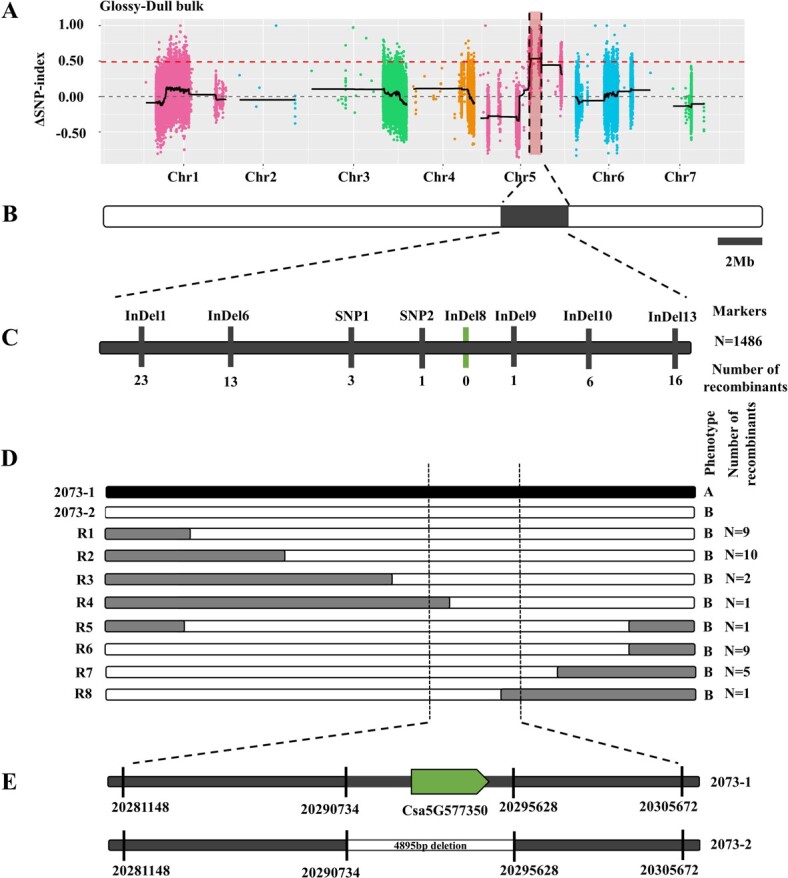
Fine mapping of the *D* locus. (A) *Δ*SNP index plot. *Δ*SNP index = SNP index (Glossy) − SNP index (Dull). The red dashed line represents the threshold (0.49) of *Δ*SNP index. The colored dots represent the calculated *Δ*SNP index value of each SNP locus and black lines represent the *Δ*SNP index after fitting. (B) Black box represents the rough positioning result of *D*. (C) Distribution of InDel and SNP markers in the rough mapping interval of *D*. (D) Genotyping of 38 recombinants. Black, white, and gray segments indicate regions homozygous for 2073-1, regions homozygous for 2073-2, and heterozygous regions, respectively. (E) *Csa5G577350* was present in the dull-fruited 2073-1 but absent in 2073-2 due to a 4895-bp deletion in the *D* mapped interval.

In BSA-seq, *Δ*SNP index is a useful parameter reflecting genotypic frequency differences of single-nucleotide polymorphisms (SNPs) between the two bulks [24, 25]. The stronger the association between SNP and target trait, the closer the *Δ*SNP index is to 1. Based on the theoretical separation ratio of our segregating population, the calculated association threshold was 0.667. But judging from the results there were no regions that exceeded the theoretical threshold. To make full use of the data, the threshold was lowered to find more likely localization regions. Using the 99th percentile of the fitted *Δ*SNP index, which was 0.49, we obtained only one region spanning 3.59 Mb on chromosome 5 between 17 358 895 and 20 945 460 in 9930v2.0 that was strongly associated with the glossy phenotype (Fig. 2A and B). Within this region, we developed eight available InDel and SNP markers for fine mapping (Fig. 2C). Eventually, we detected 38 recombinants among 366 recessive populations, which belonged to eight categories (R1–R8) (Fig. 2D). Based on their genotypic and phenotypic data, recombinant types R1, R2, R3, and R4 placed the *D* locus downstream of marker SNP2, while recombinant types R6, R7, and R8 indicated that the *D* locus mapped upstream of marker InDel9. In summary, the *D* locus was finally narrowed down to a 24.5-kb region defined by markers SNP2 and InDel9.

### *CsDULL*, a C_2_H_2_ transcription factor, is the candidate gene for the *D* locus

In the 9930v2.0 genome, only one gene, *Csa5G577350*, was predicted in this 24.5-kb region that encodes a C_2_H_2_-type transcription factor (Fig. 2E). We tried to clone the genomic DNA sequences of *Csa5G577350* from 2073-1 and 2073-2. We were able to clone this gene from the dull-fruited 2073-1, but failed to clone it from glossy-fruited 2073-2. We speculate that a presence-absence variation (PAV) variant may exist within this interval of the 2073-2 genome, and resequencing data verified our speculation. Subsequently, we designed a codominant marker InDel-DULL to demonstrated the presence of this PAV by PCR and agarose gel electrophoresis (Supplementary Data Fig. S2A). Combining the resequencing data and multiple sequencing results, we determined a deletion of 4895 bp (20 290 734-20 295 628) in the 2073-2 genome, including the promoter and coding sequence of *Csa5G577350* (Fig. 2E; Supplementary Data Fig. S2B). This suggests that *Csa5G577350* (hereafter referred to as *CsDULL*) is the best candidate gene for the *D* locus.

To further validate that the 4895-bp deletion is responsible for the glossy fruit phenotype, the codominant marker InDel-DULL was used to test 42 cucumber lines with dull or glossy fruits (Supplementary Data Table S2). When the 4895-bp deletion was present in the tested line, a 401-bp band could be detected with the codominant marker InDel-DULL, and otherwise a 5296-bp band could be detected. The results showed that the 4895-bp deletion was absent in all 21 dull-fruited lines but existed in all 21 glossy-fruited lines (Supplementary Data Fig. S3). This strongly indicated that the 4895-bp deletion is closely related to the glossiness trait of cucumber fruits and that Csa5G577350 is the best candidate gene for the *D* locus.

We aligned deduced amino acid sequences of homologous DULL proteins from cucumber, melon, and other selected species (potato, *Arabidopsis*, and grape) using MEGA-X software. All these proteins contained a highly conserved amino acid sequence, QALGGH, that is unique to plant C_2_H_2_ zinc finger proteins (Supplementary Data Fig. S4A). In addition, all homologs except *Arabidopsis* SUP also contained two highly conserved motifs, LKLFGF/I and G/SLDLHL, at their N terminus and C terminus, respectively (Supplementary Data Fig. S4A). Interestingly, we found that the conserved motif of CsDULL at the C terminus seems to be a variant EAR motif rich in leucine, which is designated as the ERF-associated amphiphilic repression (EAR) motif and always contains a conserved consensus sequence of LxLxL or DLNxxP″ [26, 27]. Phylogenetic analysis found that, except for SUP, all others were clustered based on the established evolutionary status of flowering plants (Supplementary Data Fig. S4B), suggesting possible conservation of functions of these DULL homologs.

### Spatial–temporal expression patterns of *CsDULL*

**Figure 3:**
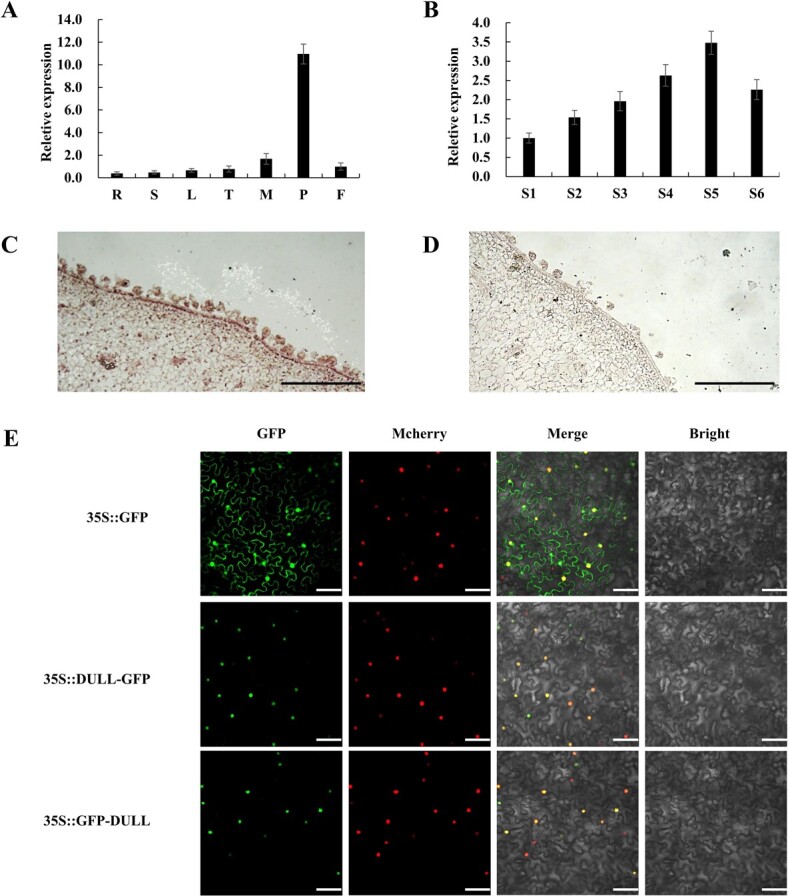
Characterization of *CsDULL* candidate gene. (A, B) qRT–PCR analysis of *CsDULL* in different tissues (A) and fruit peel at different developmental stages (B) in dull-fruited 2073-1. R, root; S, stem; L, leaf; T, tendril; M, male flower; P, peel; F, flesh. Error bars represent the mean ± standard deviation (*n* = 3). (C, D) mRNA *in situ* hybridization of Cs*DULL* in 2073-1 fruits at stage 2 with antisense (C) and sense (D) probes. (E) Subcellular localization of the CsDULL–GFP and GFP–CsDULL fusion proteins in *N. benthamiana* leaves. Separate GFP driven by the 35S promoter was used as a control. NF-YA4-mCherry was used as a nuclear marker. Scale bars = 500 μm (C, D) or 50 μm (E).

Tissue type-specific gene expression is essential for plants to perform diverse and specialized functions in different tissues. Therefore, we evaluated the expression of *CsDULL* in different tissues and organs of 2073-1, and found that it was expressed mainly in fruit peel (Fig. 3A). We further examined its expression at different fruit developmental stages. *CsDULL* was expressed at different stages of fruit development, especially at stages 3, 4, and 5, when cuticle development is very active (Figs 3B and 1C), implying an intrinsic link between the two. The mRNA *in situ* hybridization also confirmed that *CsDULL* was highly expressed in fruit peel (Fig. 3C and D). These data further support the idea that *CsDULL* is a good candidate for the *D* locus.

*CsDULL* was predicted to encode a C_2_H_2_-type transcription factor. We reason that CsDULL protein should be localized in the nucleus to play a transcriptional regulatory role. We conducted a subcellular localization assay for CsDULL. Transient expression of CsDULL-GFP and GFP-CsDULL fusion proteins in tobacco epidermal cells indeed confirmed that the CsDULL is exclusively localized in the nucleus (Fig. 3E).

### CsDULL regulates the expression of genes associated with cuticle

**Figure 4:**
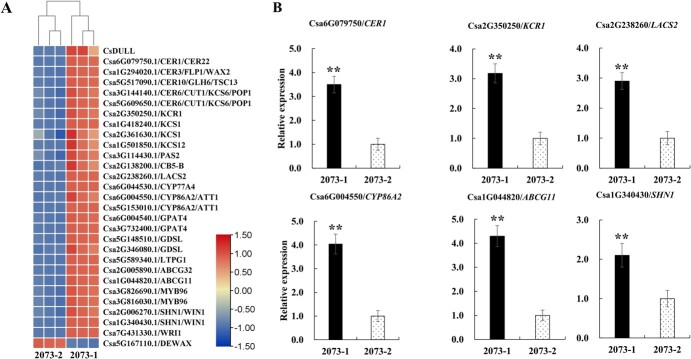
RNA-seq analysis between 2073-1 and 2073-2 NILs. (A) Heat map of cuticle-related DEGs in 2073-1 and 2073-2 in three functional categories. The colored bar on the right represents the fold change after normalization. (B) qRT–PCR validation of six DEGs from RNA-seq with peel samples of 2073-1 and 2073-2 at stage 5. Error bars represent the mean ± standard deviation (*n* = 3). The double asterisks indicates a significant difference (*P* < .01; *t*-test).

In a number of plant species, cutin and wax on the epidermis of above-ground organs have been shown to affect glossiness [18–23]. The temporal and spatial expression patterns of *CsDULL* were highly correlated with cutin and wax accumulation in the fruit epidermis (Figs 3A and B and 1C), and we speculate that CsDULL may affect fruit glossiness by regulating the expression of cuticle-related genes. To provide evidence for this, we conducted RNA-seq of 2073-1 (*DULL*) and 2073-2 (*dull*) in stage 5 fruit peels. The FPKM (fragments per kilobase of transcript per million mapped reads) values of three biological replicates of each sample were highly correlated, indicating high quality of the RNA-seq data (Supplementary Data Fig. S5A). We identified 3973 differentially expressed genes (DEGs) in the transcriptomes of the two lines, including 1839 up- and 2134 downregulated DEGs (Supplementary Data Fig. S5B, Supplementary Data Table S3). KEGG (Kyoto Encyclopedia of Genes and Genomes) analysis showed that the up- and downregulated genes in the glossy fruit peel of 2073-2 affect a variety of metabolic pathways, including cutin and wax biosynthesis, fatty acid biosynthesis, fatty acid metabolism, plant–pathogen interaction, plant hormone signaling, phenanthrene biosynthesis, and flavonoid biosynthesis (Supplementary Data Fig. S5C and D). Notably, 28 DEGs were significantly downregulated in the peel of 2073-2 and are involved in the biosynthesis, transmembrane transportation, and transcriptional regulation of cutin and wax; their biological functions have all been demonstrated in *Arabidopsis* (Fig. 4A). We validated expression patterns of six DEGs in RNA-seq with qRT–PCR, including *CER1* (ECERIFERUM 1), *KCR1* (BETA-KETOACYL REDUCTASE 1), *LACS2* (LONG-CHAIN ACYL-COA SYNTHETASE 2), *CYP86A2* (CYTOCHROME P450, FAMILY 86, SUBFAMILY A, POLYPEPTIDE 2), *ABCG11* (ATP-BINDING CASSETTE G11), and *SHN1*/*WIN1* (SHINE 1/WAX INDUCER 1), which revealed high consistency between the data sets (Fig. 4B). The results indicate that *CsDULL* may affect the glossiness of cucumber fruits by regulating cuticle-related genes.

### *CsGPAT4* and *CsLTPG1* are possible targets of CsDULL

**Figure 5:**
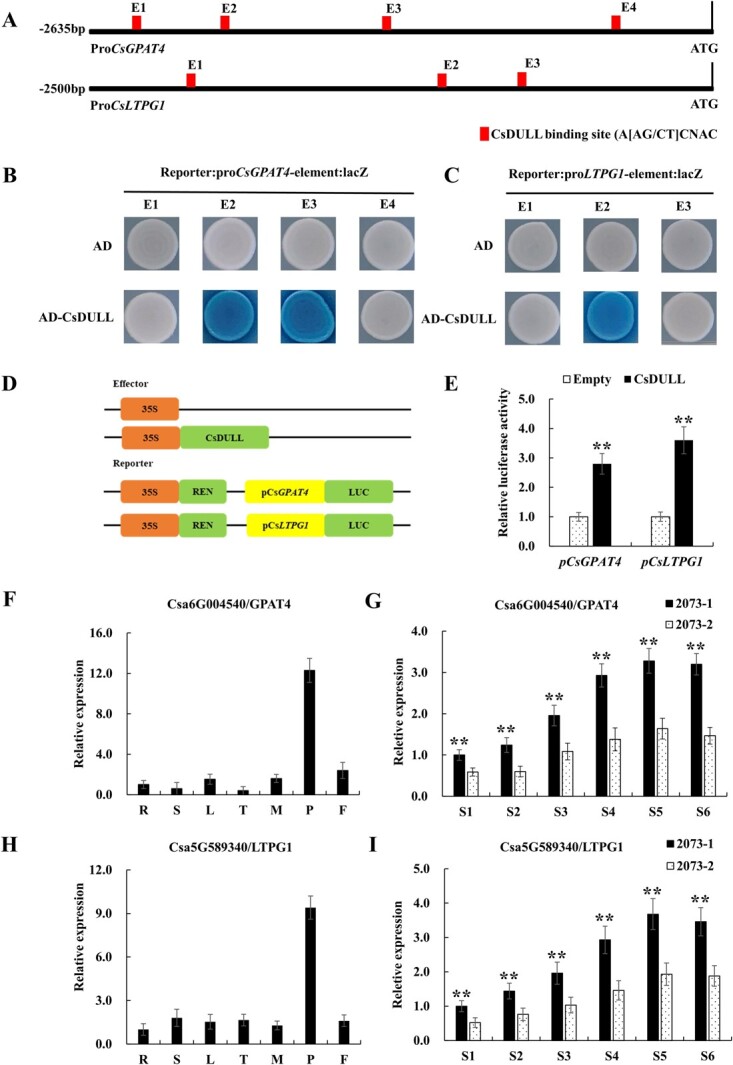
CsDULL binds directly to *CsGPAT4* and *CsLTPG1* to activate their expression. (A) Schematic diagram of the putative CsDULL binding sites (red boxes) in the promoters of *CsGPAT4* and *CsLTPG1*. (B, C) Yeast one-hybrid assays suggest that CsDULL can bind directly to the promoters of *CsGPAT4* and *CsLTPG1*. (D, E) Dual-luciferase reporter assays suggest that CsDULL can activate the expression of *CsGPAT4* and *CsLTPG1* in tobacco epidermal cells. (F, G, H, I) qPCR analysis of *CsGPAT4* and *CsLTPG1* showed that their expression patterns were highly consistent with *CsDULL*. The plant material used in F and H is 2073-1. Error bars represent the mean ± standard deviation (*n* = 3).

*Arabidopsis GIS3* (GLABROUS INFLORESCENCE STEMS 3) is the homolog of *CsDULL* that affects trichome initiation by directly binding to the A[AG/CT]CNAC sequence on the *GIS* and *GIS2* promoters, and this sequence is also a reported binding site of C_2_H_2_ zinc finger proteins [28–30]. We examined promoter regions of 28 DEGs from RNA-seq for the presence of the A[AG/CT]CNAC motif, which was found in 15 DEGs (Supplementary Data Table S4). We selected two such DEGs, *CsGPAT4* (GLYCEROL-3-PHOSPHATE SN-2-ACYLTRANSFERASE 4) and *CsLTPG1* (GLYCOSYLPHOSPHATIDYLINOSITOL-ANCHORED LIPID PROTEIN TRANSFER 1) for further investigation. There were four and three A[AG/CT]CNAC motifs in the promoter of *CsGPAT4* and *CsLTPG1*, respectively (Fig. 5A). To investigate if CsDULL was able to bind to these motifs, we conducted yeast one-hybrid (Y1H) and dual-luciferase (dual-LUC) assays and found that CsDULL was indeed able to target the promoters of *CsGPAT4* and *CsLTPG1* to promote their expressions (Fig. 5B, C, E). We examined the spatiotemporal expression of the two target genes by qRT–PCR. The results showed that their expression patterns were highly consistent with *CsDULL* (Fig. 5F, G, H, I). These data suggested that *CsGPAT4* and *CsLTPG1* may be the direct targets of CsDULL.

## Discussion

### Fruit glossiness is an important trait with complex inheritance modes

Among a number of horticulturally important traits affecting fruit external quality in cucumber, fruit glossiness seems to be the most complex. This could be mainly reflected in three aspects. First, this trait has a different inheritance mode, which could be dominant (the *D* locus) [3–5, 8–11] or recessive (the *G* locus) [15]. More interestingly, both D and G loci have been mapped very close to each other on chromosome 5. While we cannot exclude the possibility that the *D* and *G* loci are actually allelic variants, it is not known if genetic backgrounds may also contribute to the observed inconsistencies. Second, at least seven fruit epidermal feature-related genes have been mapped in the same cluster as the *D* locus, including *Tu*, *u*, *H*, *ss*, *Te*, *Pe*, and *Fr* [2, 12, 14]. Third, the degree of fruit glossiness is influenced by other factors, such as fruit bloom and environmental conditions [31, 32]. Fruit bloom, also known as wax powder, is a gray–white frosty mixture covering the surface of cucumber fruit, whose presence will greatly reduce the glossiness of fruit. In addition, years of breeding practice have shown us that environmental factors such as temperature, light, and water can also affect the glossiness of cucumber fruit. In the present study, we developed near-isogenic lines for the *D* locus, which greatly helped reliable phenotyping and map-based cloning of this important fruit appearance quality trait.

### *CsDULL* controls the glossiness of cucumber fruit by regulating the expression of cuticle-related genes

The plant cuticle is composed of cutins and waxes. It is a layer of lipophilic compounds deposited on the polysaccharide layer of the epidermal cell wall and covers the outermost layer of the above-ground parts of almost all plants [33–36]. Plant cuticle is of great significance in preventing water loss, pathogen invasion, insect encroachment, ultraviolet damage, and organ fusion [35, 36]. In addition, studies have shown that the chemical composition, content, and morphological structure of the cuticle of the plant epidermis can affect its glossiness [18–23]. In this study, we found that the water loss rate and epidermal permeability of the 2073-2 glossy fruits were significantly increased (Fig. 1G, H, I, K). We speculate that glossy fruits may have a defect in the cuticle that may cause the glossy fruit phenotype. The Sudan IV staining results seemed to support this (Fig. 1C and D). To understand the mechanism of *CsDULL*-regulated cuticle development, it is necessary to identify its downstream target genes. We attempted to identify these genes from RNA-seq data based on two criteria: first, their orthologs in *Arabidopsis* have a clear biological function in cuticle formation, and second, the genes have a similar spatiotemporal expression pattern to *CsDULL*. We identified 28 possible target genes of CsDULL from RNA-seq data. The homologues of these genes in *Arabidopsis* encode key catalytic enzymes, transporters, and transcription factors, which are widely involved in wax and cutin biosynthesis, transmembrane transport, and transcriptional regulation [35, 37]. We further reasoned that the target gene promoters may contain the characteristic A[AG/CT]CNAC sequence motifs for C_2_H_2_-type zinc finger transcription factors. Indeed, 15 of the 28 DEGs contained the motifs, including *CsGPAT4* and *CsLTPG1. CsGPAT4* encodes a glycerol-3-phosphate acyltransferase, which catalyzes the formation of lysophosphatidic acid (LPA) from the lipid acylation of glycerol-3-phosphate (G3P); it is a rate-limiting enzyme in the lipid biosynthesis pathway [38]. In *Arabidopsis*, *GPAT4* is involved in the biosynthesis of cutin in leaves and stems, which in turn affects the drought and disease resistance of the plant [39, 40]. *CsLTPG1* encodes a glycosylphosphatidylinositol (GPI)-anchored lipid transporter, a protein with lipid-binding activity that frequently accumulates in plant epidermal cells and can directly or indirectly promote the export or accumulation of cuticular wax [41, 42]. Y1H and dual-LUC assays showed that CsDULL can bind to the promoter sequences of *CsGPAT4* and *CsLTPG1* and promote their expression (Fig. 5B–E), which supports a critical role of CsDULL in regulating cuticle development and fruit glossiness.

### *CsDULL* may be responsible for both fruit glossiness and wart formation

In this study, through fine genetic mapping in NIL-derived segregating populations, we successfully narrowed down the *D* locus to a 24.5-kb region that contained only one candidate gene, *Csa5G577350*/*CsDULL*, which encodes a C_2_H_2_-type zinc finger transcription factor (Fig. 2). Interestingly, *Csa5G577350* has also been shown to be the candidate gene for the *Tu* (tuberculated fruit) [16]. Yang *et al*. [16] found that, compared with warty fruit, smooth fruits (no warts) contain a 4888-bp deletion with complete loss of *Csa5G577350* [16]. This is very similar to the finding in this study that a 4895-bp deletion is present in the glossy-fruited line (2073-2), including *CsDULL* (Fig. 2D). Since the glossy fruit of 2073-2 was also wart-free and the dull fruit of 2073-1 has obvious tubercules (Fig. 1A and B), we carefully examined the association of the two traits among 366 glossy F_2_ plants with glossy fruits. Indeed, we found all glossy fruits also did not have any tubercules (Supplementary Data Table S5). We also examined the lines used by Yang *et al*. [16] and found that all fruits without tubercules or with small tubercules had a glossy phenotype, while all fruits with large tubercules showed a dull phenotype, which was consistent with the observations in Yang *et al*. [3, 16].

Yang *et al*. [16] suggested that *Tu* regulates wart formation in cucumber through upregulation of cytokinin (CTK) hydroxylase-like genes *Csa5G644580* and *Csa5G224130* to promote CTK biosynthesis, which stimulates cell division and leads to fruit tubercule formation [16]. In another study, *CsTS1* (*TUBERCULE SIZE 1*), a direct target gene of *Tu*, could regulate fruit tubercule size through the indole acetic acid (IAA) signal pathway [43]. Coincidentally, in the present study, KEGG analysis showed that DEGs in the peels of 2073-1 and 2073-2 NILs not only affected various metabolic pathways involved in cuticle biosynthesis, but also affected plant hormone signaling pathways including CTK and IAA (Supplementary Data Fig. S3C and D, Supplementary Data Table S3). After careful analysis of multiple results, we suggest that *D* and *Tu* encode the same gene, and this result could well explain the puzzle that has plagued cucumber breeders for nearly a century.

The close linkage of the *D* and *Tu* loci has been known for a long time [12]. The present study and Yang *et al*. [16] present evidence for the first time that they may actually belong to the same loci. That is, this C_2_H_2_ zinc finger transcription factor may regulate both fruit glossiness and wart formation. It is also reasonable to speculate that this transcription factor may also underlie the dominant gene *G* locus for fruit glossiness as described by Miao *et al*. [8]. However, additional work is clearly needed to substantiate these observations.

In summary, we identified a C_2_H_2_-type zinc finger protein, CsDULL, in this study, which is responsible for the *D* locus. Our findings will provide a genetic and theoretical basis for comprehensive improvement of cucumber fruit appearance quality and are of great value for future cucumber breeding.

## Materials and methods

### Plant materials and phenotyping fruit glossiness

The cucumber lines 2073-1 (P_1_, dull fruit) and 2073-2 (P_2_, glossy fruit) used in this study are a pair of near-isogenic lines (NILs) at the *D* locus and were isolated from a high-generation inbred population [17]. More concretely, 2073-1 is an inbred line selected from the F_9_ population whose female and male parent are DeltaStar (P_1_, a commercial variety of the Rijk Zwaan company) and 3461 (P_2_, a high-generation inbred line developed by our laboratory), respectively. In addition, 2073-2 is a glossy mutant isolated from selfed progenies of 2073-1. The development process is shown in Supplementary Data Fig. S6. Seeds of the two lines are available upon request. To investigate inheritance and perform fine genetic mapping, we developed F_1_, F_2_, BC_1_P_1_, and BC_1_P_2_ populations from crosses among 2073-1 (dull) and 2073-2 (glossy) plants (details in Table 1). Plants of these materials were grown in Six Golden Rings Agricultural Park of Changping, Beijing (NILs and F_1_, 2017) or Qingxian Experimental Station at Langfang in Hebei Province (all segregating populations, 2018). Fruits were qualitatively scored as either dull or glossy at stage 5, which was ~15 days after flowering. For accuracy, only three well-developed fruits set on nodes 8–16 of a plant were kept for scoring.

### Water loss determination and toluidine blue assays

To evaluate the water loss ratio of fruit peel, 15 stage 5 fruits were collected from each of the NILs 2073-1 and 2073-2 NILs, and the stalk area of each fruit was sealed with a tape to prevent non-peel water loss. Fruits were stored at 37°C and weights were determined every 8 hours for 2 days. TB staining was performed to identify defects in the ovary or fruit cuticle according to the method described by Tanaka *et al*. [44]. The samples used for TB assay analysis were whole ovaries of stage 1 and stage 2, and a rectangular piece of peel from stage 3, 4, and 5 fruits. An aqueous solution of 0.05% (w/v) TB (Sigma, USA) that had been filtered through a fiber medium filter (pore diameter, 0.2 μm) was poured directly into the staining vessel containing the above samples until the samples were submerged. After 10 minutes the TB solution was removed and the samples were gently washed with water to remove residual TB, followed by photography with a high-definition camera.

### Fine genetic mapping and identification of candidate gene for the *D* locus

BSA was used to determine sub-chromosomal locations of the *D* locus. Genomic DNAs were extracted from the two NILs and from F_2_ plants using the CTAB method. Two bulks, dull and glossy, were constructed by pooling equal amounts of genomic DNA from 50 dull and 50 glossy F_2_ plants, respectively. High-throughput genome sequencing and data analysis of the two bulks and two parental lines was conducted by Beijing Biomarker Biotechnology Corporation (http://www.biomarker.com.cn/). Processing of raw reads, DNA calling, calculation of genome-wide SNP frequency and *Δ*SNP were performed with BWA and GATK software programs [45, 46] using 9930v2.0 as the reference genome (http://cucurbitgenomics.org/).

BSA placed the *D* locus in a 3.59-Mb region on chromosome 5. For fine mapping, InDel and SNP markers within this region were developed using the procedures described in Xu *et al*. [47]. Several rounds of marker discovery and recombinant identification using F_2_ plants located the *D* gene in a 24.5-kb region flanked by SNP2 and InDel9. Information on molecular markers for fine mapping is provided in Supplementary Data Table S6. All primers were synthesized by Shenggong Biotechnology Corporation (https://www.sangon.com/).

### Subcellular localization of CsDULL

Subcellular localization of CsDULL followed the procedure described by Nie *et al*. [48]. In brief, the full-length coding sequence of CsDULL without the stop codon was cloned from cDNA of 2073-1 and inserted in the 35S::GFP expression vector (owned by our laboratory) to form a CsDULL–GFP fusion construct. NF-YA4-mCherry was used as a nuclear marker. Subsequently, we co-transformed the fusion protein and nuclear marker into *Agrobacterium* strain GV3101, which was used to infect the epidermal cells of *Nicotiana benthamiana* leaves. After 2–3 days, GFP fluorescence and mCherry fluorescence were detected under a laser confocal microscope at an excitation wavelength of 488 and 587 nm, respectively. This experiment was performed three times. Information on primers used in this experiment is provided in Supplementary Data Table S6.

### RNA extraction, RT–PCR and qRT–PCR assays

The spatial–temporal expression of *CsDULL* and various genes was investigated. Total RNAs from different tissues or organs. cDNA synthesis, RT–PCR and qRT–PCR followed the manufacturers’ instructions at Tiangen Biotechnology Corporation (https://www.tiangen.com/) and Zhuangmeng Biotechnology Corporation (http://www.zomanbio.com/). For each treatment, there were three biological replicates and three technical replicates. The *β-actin* gene was used as internal reference in qRT–PCR.

### Yeast one-hybrid assay

The Y1H assay was performed as described previously [49]. Briefly, the full-length coding sequence of *CsDULL* was cloned with specific primers (Supplementary Data Table S6) and inserted in the effector vector pJG4–5/pB42AD (Clontech, USA). The promoter fragments (upstream of the start codon, ATG) of *CsGPAT4* (2600 bp) or *CsLTPG1* (2500 bp) were cloned into the reporter vector pLacZi (Clontech, USA). The desired pair of constructs was then co-transformed into the yeast strain EGY48 (Shanghai Weidi Biotechnology Corporation, http://www.weidibio.com/). The transformants were grown on SD-Ura-Trp medium at 30°C for 4–5 days; single clones were picked and spread on SD-Ura-Trp medium containing X-gal to observe color development.

### Dual-LUC assays

The LUC assays were performed as previously described [49]. The promoter of *CsGPAT4* and *CsLTPG1* were cloned into the expression vector pGreenII 0800-Luc (owned by our laboratory) to generate effector constructs, and the full-length coding sequence of *CsDULL* was cloned into the expression vector pGreenII 62-SK (owned by our laboratory) to generate reporter constructs (see Supplementary Data Table S6 for primer information). The effector and reporter vectors were then co-transformed into the epidermal cells of leaves of 4-week-old *N. benthamiana* seedlings. After 2–3 days, firefly luciferase and *Renilla* luciferase activities were detected using a commercial kit (Promega, Beijing, China). A construct without effector was used as negative control. Each LUC assay was repeated eight times.

## Acknowledgements

This study was supported by the National Natural Science Foundation of China (32172572), the Project of Beijing Agricultural Innovation Consortium (BAIC01), and the Construction of Beijing Science and Technology Innovation and Service Capacity in Top Subjects (CEFF-PXM2019_014207_000032). Heartfelt thanks to all the people who have ever helped me in this paper. Special thanks to professor Y.W. for his great efforts in proofreading this article.

## Author contributions

H.R. and X.L. designed and supervised the study. X.L.Z. wrote the draft and created the figures and tables. H.R., Y.Q.W., and X.L. revised the manuscript and figures. H.W., Z.Z., L.S., X.Z., R.W., C.L., and Y.W. proofread the manuscript. X.Z. agreed to serve as the author responsible for contact and to ensure communication. All authors reviewed and approved this submission.

## Data availability

The raw data are accessible at NCBI under the BioProject PRJNA824261.

## Conflict of interest

The authors declare that they have no conflict of interest.

## Supplementary data

Supplementary data is available at *Horticulture Research* online.

## Supplementary Material

Web_Material_uhac146
