# Supplementary material for: The fruit glossiness locus, dull fruit (D), encodes a C2H2-type zinc finger transcription factor, CsDULL, in cucumber (Cucumis sativus L.)
Source: Hortic Res. 2022 Jul 2;9:uhac146. doi: 10.1093/hr/uhac146 (PMC9437717; doi:10.1093/hr/uhac146)
Supplement: Web_Material_uhac146 [file web_material_uhac146.docx]

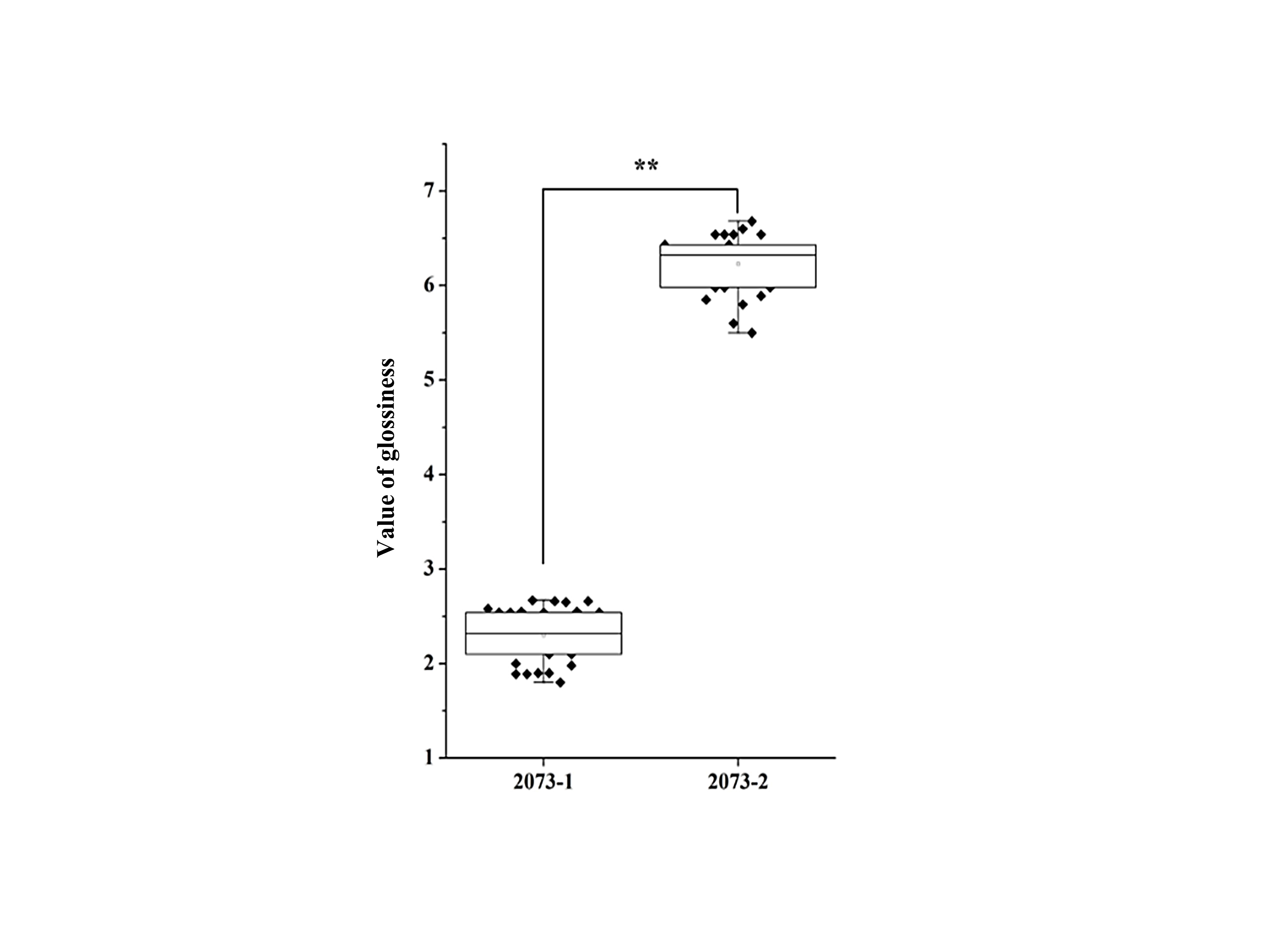


**Fig. S1: Glossiness values of fruits at stage5 for 2073-1 and 2073-2.**


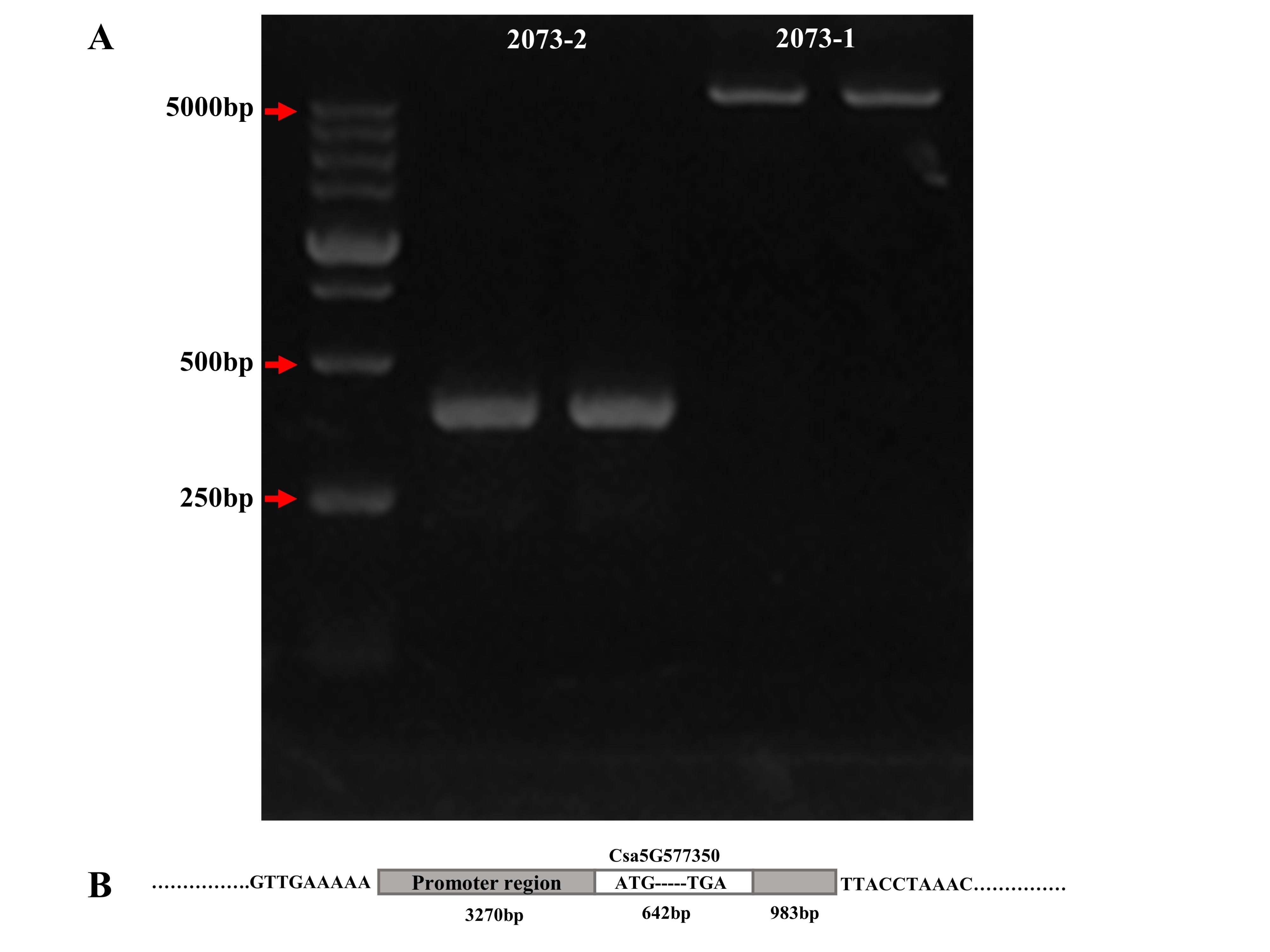


**Fig. S2: Results of PCR with the co-dominant marker InDel-DULL in 2073-1 and 2073-2.**


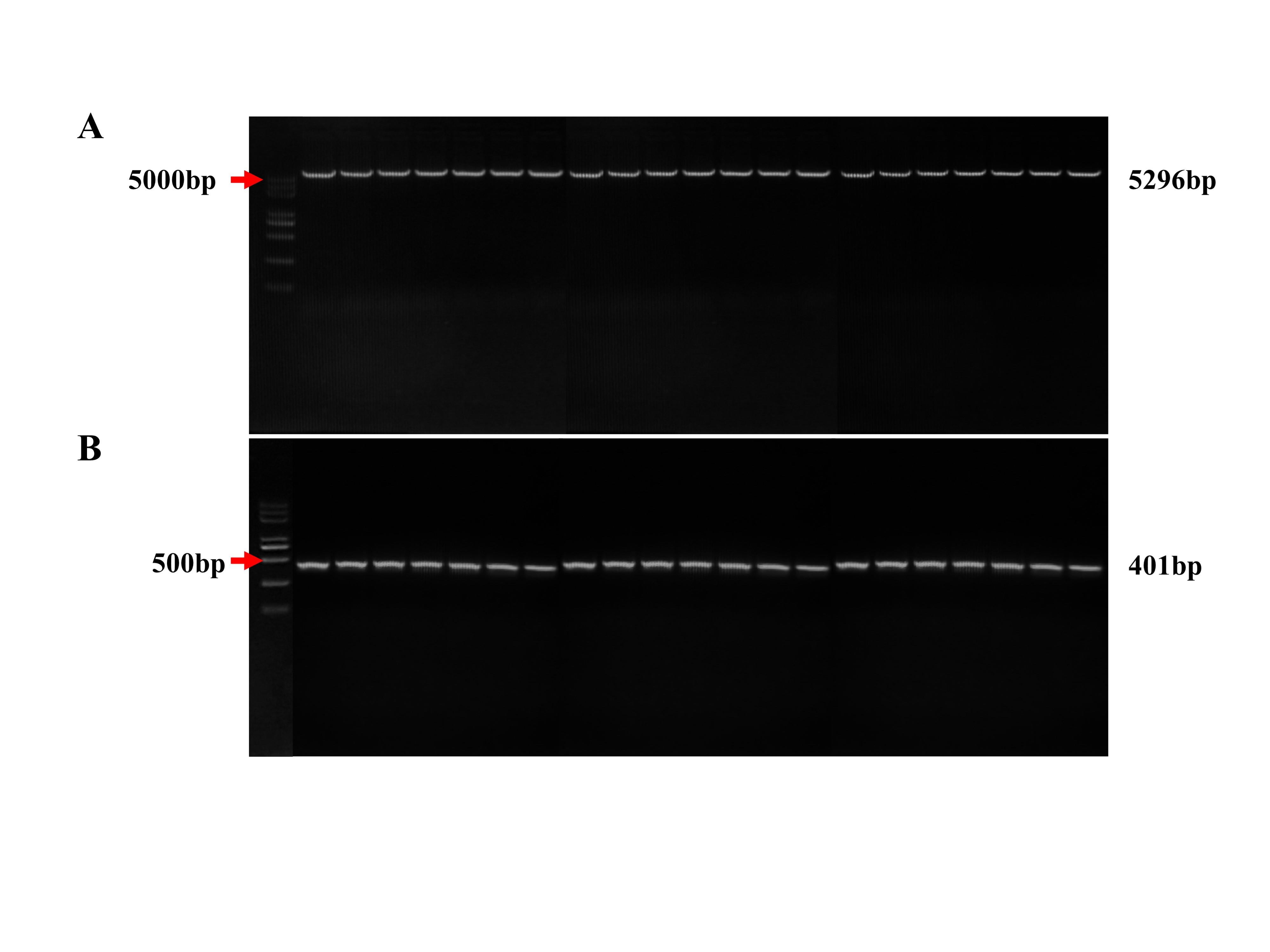


**Fig. S3: Results of PCR with the co-dominant marker InDel-DULL in 42 cucumber lines.** In Fig A, from left to right, the high generation inbred line of each lane tested is 2073-1~D20, respectively. In Fig B, from left to right, the high generation inbred line for each lane tested is 2073-2~G20, respectively.


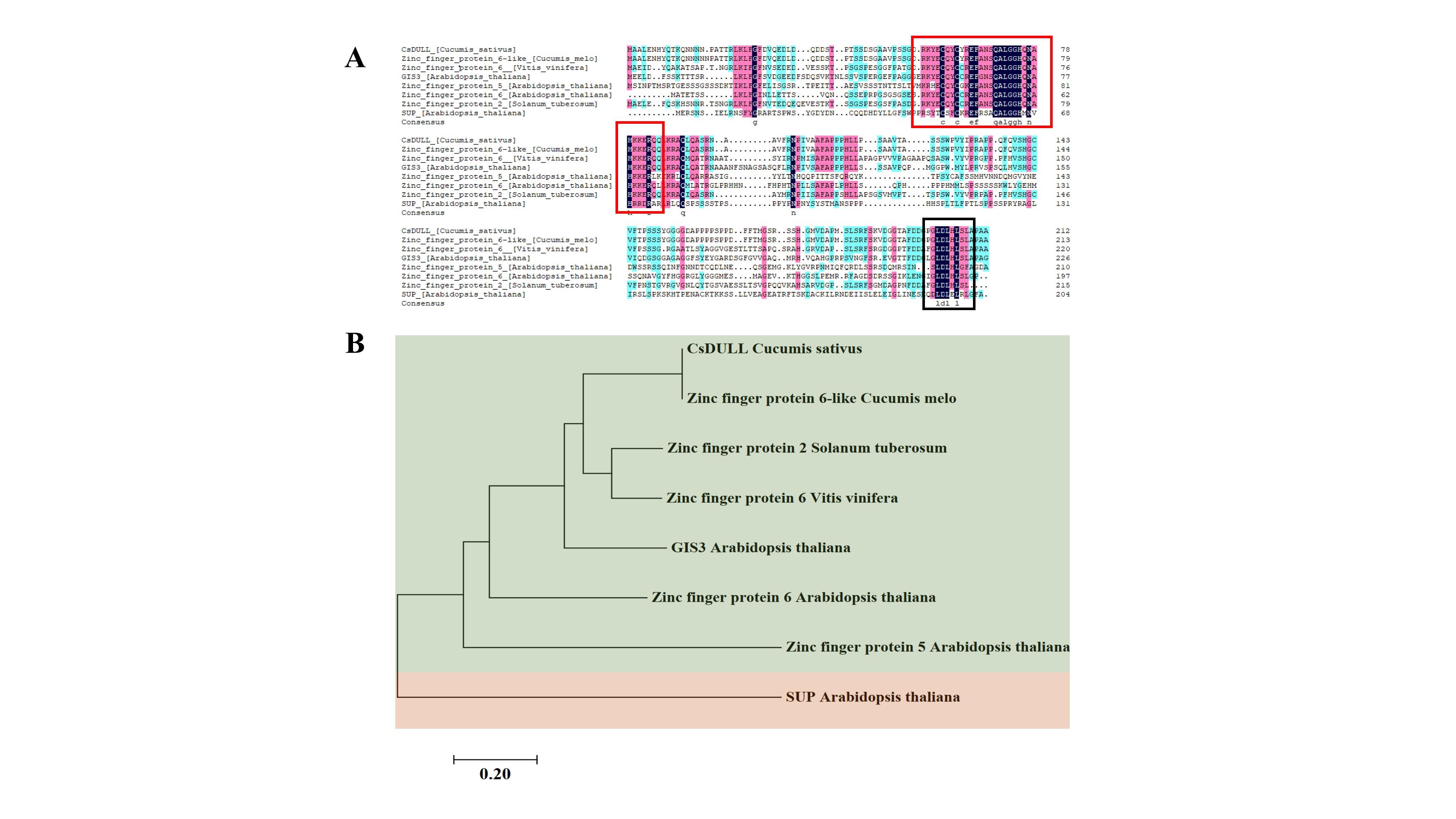


**Fig. S4: Phylogenetic tree analysis and amino acid sequence alignment. A.** Amino acid sequence alignment of CsDULL related proteins. **B.** Phylogenetic tree analysis of CsDULL related proteins.The red box indicates the C_2_H_2_ zinc finger motif and the black box indicates the EAR-like motif.


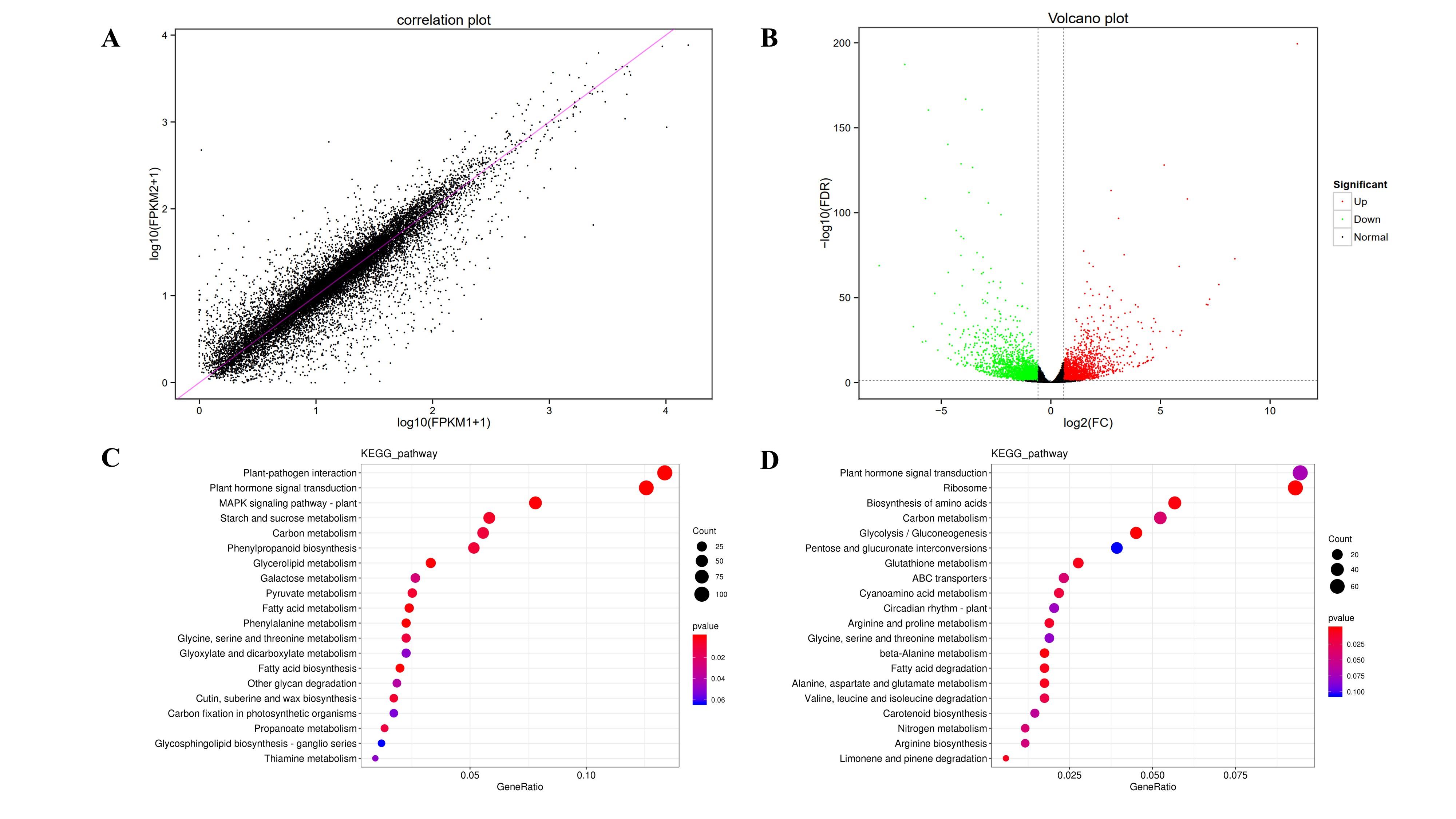


**Fig. S5: The analysis of RNA-seq data**. **A.** Expression correlation plot for all genes within RNA-seq groups. Each dot in the plot represents a gene, and the more concentrated the points are near the diagonal line, the stronger the correlation between gene expressions in the two subgroups, and the dots off the diagonal line represent DEGs. **B.** Volcano graph of differentially expressed genes. The green dots in the graph represent down-regulated DEGs, the red dots represent up-regulated DEGs, and the black dots represent non-differentially expressed genes. **C.** KEGG analysis of down-regulated DEGs in RNA-seq of 2073-1 VS 2073-2. **D.** KEGG analysis of up-regulated DEGs in RNA-seq of 2073-1 VS 2073-2.


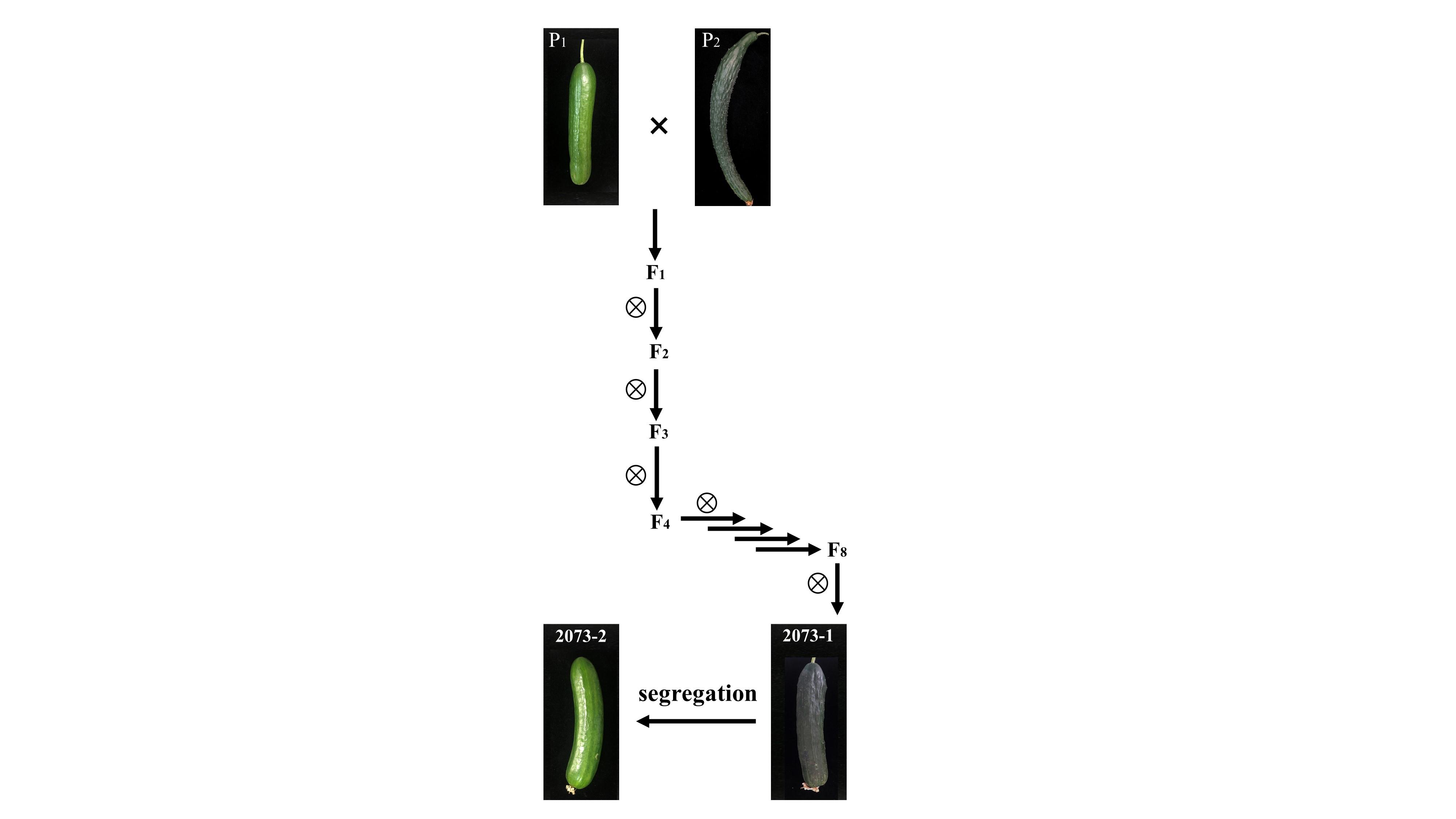


**Fig. S6: The development process of near-isogenic lines 2073-1 and 2073-2.** P_1_, DeltaStar (a commercial variety of RIJK ZWAAN company); P_2,_ 3461 (a high-generation inbred line developed by our laboratory).
